# Operon Conservation and the Evolution of *trans*-Splicing in the Phylum Nematoda

**DOI:** 10.1371/journal.pgen.0020198

**Published:** 2006-11-24

**Authors:** David B Guiliano, Mark L Blaxter

**Affiliations:** Institute of Evolutionary Biology, University of Edinburgh, Edinburgh, United Kingdom; National Institute of Genetics, Japan

## Abstract

The nematode Caenorhabditis elegans is unique among model animals in that many of its genes are cotranscribed as polycistronic pre-mRNAs from operons. The mechanism by which these operonic transcripts are resolved into mature mRNAs includes *trans*-splicing to a family of SL2-like spliced leader exons. SL2-like spliced leaders are distinct from SL1, the major spliced leader in C. elegans and other nematode species. We surveyed five additional nematode species, representing three of the five major clades of the phylum Nematoda, for the presence of operons and the use of *trans*-spliced leaders in resolution of polycistronic pre-mRNAs. Conserved operons were found in *Pristionchus pacificus, Nippostrongylus brasiliensis, Strongyloides ratti, Brugia malayi,* and *Ascaris suum.* In nematodes closely related to the rhabditine *C. elegans,* a related family of SL2-like spliced leaders is used for operonic transcript resolution. However, in the tylenchine S. ratti operonic transcripts are resolved using a family of spliced leaders related to SL1. Non-operonic genes in S. ratti may also receive these SL1 variants. In the spirurine nematodes B. malayi and A. suum operonic transcripts are resolved using SL1. Mapping these phenotypes onto the robust molecular phylogeny for the Nematoda suggests that operons evolved before SL2-like spliced leaders, which are an evolutionary invention of the rhabditine lineage.

## Synopsis

The genome of the nematode worm Caenorhabditis elegans was the first of any animal to be completely sequenced. One surprising finding in this worm's genome was that about one-fifth of its genes were organised as sets of from two to eight genes expressed from the same promoter, similar to bacterial “operons.” The pre-mRNAs made from these operons are processed by an intermolecular ligation process called SL *trans*-splicing. Other animal genomes, such as the human genome or that of the fruit fly contain neither operons nor SL *trans-*splicing. In this article, Guiliano and Blaxter have investigated whether this curious facet of genome organisation is peculiar to C. elegans and close relatives by examining the genomes of a wide range of parasitic and free-living nematodes. The authors find that both operons and *trans-*splicing are present across the nematodes, and that operons evolve as other genome features do. All of the species surveyed use *trans*-splicing to resolve their multigene pre-mRNAs into single-gene mRNAs, but the details differ significantly from the process in *C. elegans.* In particular, the short piece of RNA that is attached to the beginning of operon-derived mRNAs has changed independently in many nematode groups.

## Introduction

Intermolecular ligation or *trans*-splicing of RNA molecules is a process that has been shown to occur in all eukaryotes tested [1]. One common *trans*-splicing reaction is the addition of a short exon called a spliced leader (SL) to the 5′ end of mRNAs. SLs have been identified in a variety of eukaryotes, including trypanosomatid protozoa [2], cnidaria [3,4], urochordates [5–7], rotifers [8], nematodes [9], and platyhelminthes [10–12]. The extent of SL *trans-*splicing to the 5′ end of mRNAs as well as the nucleotide sequence of the mini-exons utilised by each group are highly variable. Different proportions of mRNAs are *trans-*spliced (from 100% in trypanosomatids to <20% in platyhelminths), and SL sequences can vary within a phylum [8,10–12].

**Figure 1:**
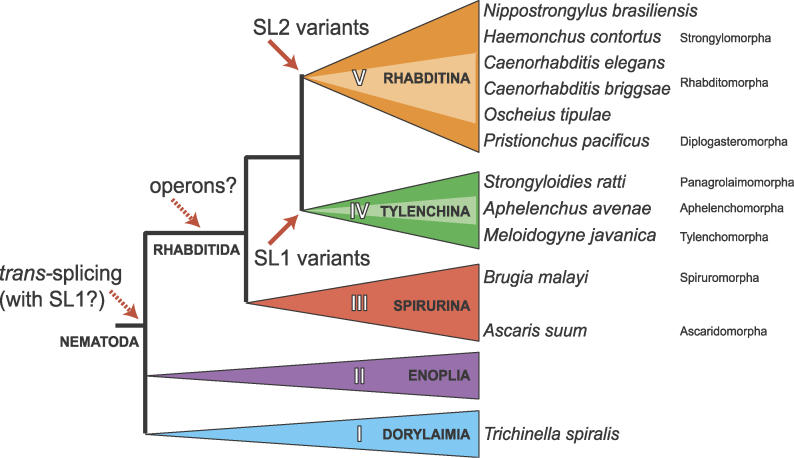
The Evolution of Operons and SL Usage through the Phylum Nematoda Operons and SL usage in *trans*-splicing have been mapped onto a phylogeny of the Nematoda illustrating the relationships of the nematodes studied based on analysis of the small subunit rRNA (adapted from [37,38]). Conserved operons have been identified in Rhabditina, Tylenchina, and Spriurina (clades V, IV, and III of [37]). While SL1 may be a synapomorphy for the phylum, SL2-like SLs are apparently restricted to the Rhabditina, as is their use in *trans-*splicing to downstream genes in operons. An independent radiation of SL1-like SLs is used for downstream gene *trans*-splicing in the Tylenchina, while the Spirurina use canonical SL1.

SL1 (a noncoding 22-nucleotide sequence) was the first SL sequence identified in nematodes [9,13]. It has been identified in all nematode species surveyed (Figure 1), suggesting that it is a molecular snyapomorphy for the phylum Nematoda [14,15]. Nematode SL *trans*-*s*plicing has been extensively studied in the model organism Caenorhabditis elegans and in the pig gut parasite Ascaris suum [16]*.* The majority of C. elegans mRNAs have SL1 *trans*-spliced to their 5′ ends. SL1 addition is mechanistically similar to standard *cis*-splicing, except that the first splice acceptor site is found upstream of the initiation ATG in the pre-mRNA. This is ligated to a donor site in a 110-nucleotide SL1 small nuclear RNA (snRNA) [17]. The biological role of *trans*-splicing is likely to be a combination of mRNA stabilisation through donation of the cap structure on the SL RNA, sanitisation of the 5′ untranslated region (UTR) of pre-mRNAs, and optimal translation via specific interactions of the SL sequence and trimethylguanosine (TMG) capped transcripts with the translation machinery [14,18,19]. The relative roles of the TMG cap structure, the presence and sequence of the SL, and the spacing of the cap and the initiation ATG has been carefully dissected in an A. suum cell-free translational system [17]. The findings suggest that the TMG and SL act synergistically to promote translation [19].

In *C. elegans,* SLs have a second role in the resolution of polycistronic mRNAs transcribed from operons [20]. Operons were discovered during attempts to define promoter elements by transgenesis. Some genes, lying immediately adjacent to each other in the same transcriptional orientation, are cotranscribed using a promoter 5′ to the upstream gene [21]. It was subsequently found that the polycistronic primary transcripts are resolved by *trans-*splicing, in a processing step that is intimately linked to polyadenylation of the upstream gene [22]. Startlingly, the majority of the SL sequences added to downstream genes in C. elegans operons are not the canonical SL1, but have distinct sequences, and are called SL2-like [20,23–25]. The nomenclature of SL2-like SLs has suffered from some confusion historically, but has been clarified by Tom Blumenthal in the C. elegans genome database WormBase (see Table S1 for a list of C. elegans SL2-like SL genes and their synonyms). In C. elegans, SL2-like SLs are derived from a family of 19 SL2-like snRNA genes dispersed across the genome, while the ~110 copies of the SL1 RNA gene are found in a tandem array with the 5S ribosomal RNA gene [18]. The 19 SL2-like genes have 13 different SL sequences at their 5′ ends, while the SL1 SL sequences are monomorphic. The SL2 *trans*-splicing complex is similar to the one that carries out SL1 *trans-*splicing, except for the presence of unique accessory proteins [26]. A C. elegans operon can be identified by the close proximity of two protein-coding genes in the same transcriptional orientation with ~100 bases between the polyadenylation site of one gene and the initiation ATG of the next. However, the C. elegans genome has yielded examples of complex, alternatively spliced variant operonic structures [27].

Genome-wide analysis using both the sequence features of operons and hybridisation of SL2-primed cDNA probes against whole-transcriptome microarrays [28] have revealed that approximately 20% of C. elegans genes are arranged in 1,054 operons of between two and eight genes. Genes in C. elegans operons are enriched for core transcriptional or translational processes, including nuclear and mitochondrial ribosomal proteins, and genes involved in RNA processing and stability [29]**.** Genes with tissue- and stage-specific expression tend not to be in operons, while genes expressed at high levels in the hermaphrodite germline are overrepresented. These features suggest that operons may be associated with genes expressed constitutively, or widely. It is important to note that different mature mRNAs derived from one operon may have very different half-lives, and thus that coexpression from a shared promoter is not necessarily associated with coordinated levels of translation and protein function. Some operons include genes that have related functions, such as the protein disulphide isomerase *pdi-1*–cyclophilin *cyn-9* operon CEOP3132 [30], but most operon gene sets have no clear functional relationship.

Analysis of the genome of a second caenorhabditid, *Caenorhabditis briggsae,* showed that both operons and SL2-like SLs are conserved in the genus. About 97% of C. elegans operons are conserved in *C. briggsae,* and C. briggsae has 18 SL2-like RNA genes, some of which encode variants compared to the C. elegans SL2-like gene set [31]. Operonic gene organisation and SL2-like SLs have been reported in two other rhabditine nematodes closely related to the caenorhabditids: in the free-living species Oscheius tipulae strain CEW1 (called Dolichorhabditis sp. or Oscheius brevesophaga in the original reports) [32] and in Pristionchus pacificus [33]. SL2-like SLs have been described in Haemonchus contortus (a sheep parasitic nematode, again closely related to C. elegans) [34]. While SL2-like sequences have proven to be highly polymorphic, SL1 appears to be largely invariant across the phylum. However, variant SL1 SLs have been described in two tylenchine species, Aphelenchus avenae [35] and Meloidogyne javanica [36] (see Figure 1 for the relationships of all nematode species mentioned) [37,38].

We are interested in the processes of genome evolution in the Nematoda [39], and thus are intrigued by the presence of *trans-*splicing, operons, and variant SL2 and SL1 genes in these taxa. Several important questions need to be addressed to further the understanding of the evolution of these processes. When did *trans*-splicing with SL RNAs arise in the phylum? When did operons arise? When did SL2-like SLs arise? Are SL2-like SLs always associated with operonic arrangements, and are operons always associated with SL2-like SLs? To approach answers to these questions, we have identified potential operons in a wide phylogenetic range of nematode species, and surveyed their pre-mRNAs and mature mRNAs for hallmarks of polycistronic transcription and alternate SL usage. Mapping our findings on the robust molecular phylogeny for the phylum [37,38], we find that operons preceded the evolution of SL2-like SLs, and that some taxa use SL1 for all *trans-*splicing, including operon-derived polycistronic pre-mRNA resolution, while others have an independent radiation of SL1-like SLs that are utilised in operonic resolution.

## Results

### Identification of Potentially Operonic Genes in a Range of Nematode Species

To identify potential nematode operons we employed a cloning by synteny approach. Potential orthologues of C. elegans operonic genes were identified in the *Brugia malayi* expressed sequence tag (EST) dataset (presented in NEMBASE [40]). Genes predicted from the B. malayi EST clusters were designated potential orthologues of the *C elegans* genes if they bore high levels of identity over the length of available protein sequence and did not have any other obvious closely related genes in either dataset (to reduce confusion with potential paralogues). We focused on operons containing C. elegans ribosomal protein genes as candidates because of their predilection for operonic organization [29]. Nearly half (49%) of 133 identified C. elegans ribosomal protein genes were found in operonic structures. In addition, in other eukaryotes surveyed, ribosomal protein genes tend to remain as single-copy genes, again reducing the chance of error due to paralogy [41]. From the initial list of candidates (see Table S3) we selected ten for testing (Table 1). For each candidate pair, primers were designed and tested against B. malayi genomic DNA. Three potential B. malayi operons were identified, orthologues of CEOP1032 *(rpl-27a/rpa-1),* CEOP1624 *(rpa-0/tct-1),* and CEOP3416 (*rpl-36*/F37C12.3). Analysis of genomic survey sequence [42] showed that the B. malayi orthologue of *rps-14,* a gene located immediately upstream of CEOP3416 in C. elegans, is conserved in the same relative location in the B. malayi genome.

**Table 1:**
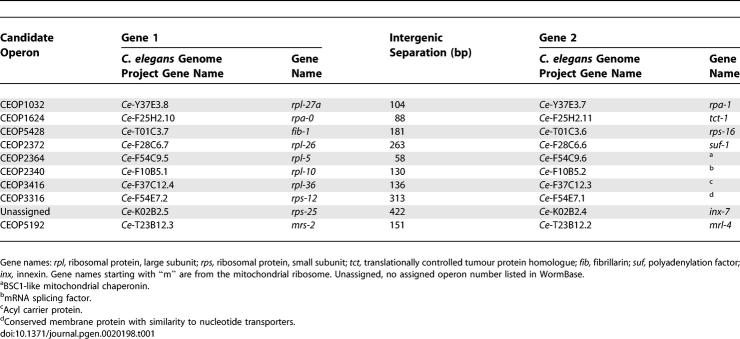
Candidate Operon Partners Identified in the C. elegans Ribosomal Proteome Gene Set and Tested in B. malayi

We surveyed four additional species for conserved operons (Figure 1): *A. suum, Strongyloides ratti* (an intestinal parasite of rats), Nippostrongylus brasiliensis (also an intestinal parasite of rats, but not closely related to S. ratti), and P. pacificus (a free-living nematode developed as a satellite model to C. elegans). In each case we were able to identify potential operonic structures for the candidate operons (Table 2). An orthologue of CEOP1032 was previously identified in the free-living rhabditid O. tipulae [29]. In addition, the operon CEOP5428 *(fib-1/rps-16)* was identified in *P. pacificus,* but is absent from B. malayi (as verified from whole-genome assembly sequence available at TIGR; http://www.tigr.org/tdb/e2k1/bma1) and was not identified in S. ratti by long-range PCR.

**Table 2:**
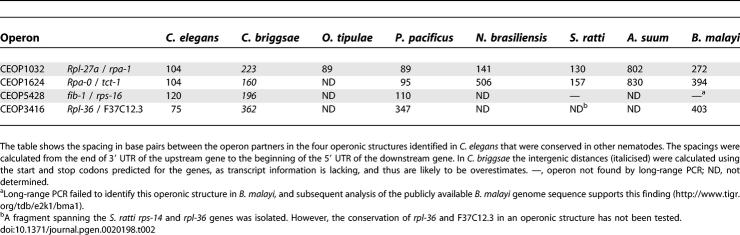
Spacing in base pairs between Genes in Conserved Operonic Structures in Nematodes

### Features of Conserved Nematode Operons

**Figure 2:**
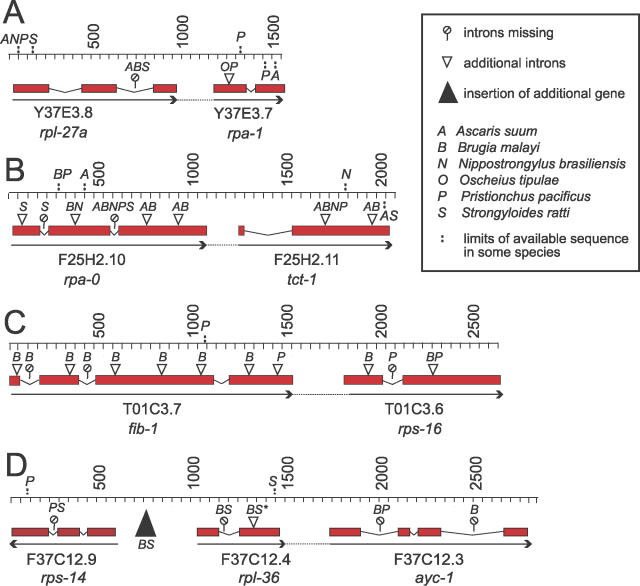
Conservation of Operonic Structures in Distantly Related Nematodes The genomic structure of four conserved nematode operons is shown. Exons are depicted with rectangles, and introns with thin lines. The gene structures and scales (in base pairs) are from the C. elegans genomic sequence; the structure of the operons and the exon/intron boundaries are conserved in *C. briggsae.* Arrows indicate direction of transcription, and the dotted lines linking arrows indicate operonic structures. The position of novel introns in other nematodes is indicated by open arrowheads. Where only a fragment of the operon has been isolated, the extents of the isolated fragment are indicated on the base scale by vertical dashed lines and the taxa thus affected by letters. The lollipop symbols indicate absence of the intron in the indicated species. (A) CEOP1032 containing *rpl-27a* and *rpa-1:* orthologues identified in *A. suum, B. malayi, N. brasiliensis, O. tipulae, P. pacificus,* and *S. ratti.* (B) CEOP1624 containing *rpa-1* and *tct-1:* orthologues identified in *A. suum, B. malayi, P. pacificus, S. ratti,* and *N. brasiliensis.* (C) CEOP5428 containing *fib-1* and *rps-16:* orthologues found in *P. pacificus.* (D) CEOP3416 containing *rpl-36* and F37C12.3 (an acyl carrier protein): orthologues found in *B. malayi, P. pacificus,* and *S. ratti.* The genomic structure surrounding CEOP3416 is also conserved. It contains four genes (F37C12.1, .2, .3, and .4) and spans a gene on the opposite strand (F37C12.14, in an intron of F37C12.1). *rps-14* is found immediately upstream, and *rps-21* one gene downstream, on the opposite strand. The filled triangle indicates the presence in B. malayi and S. ratti of an additional gene that shows similarity to C. elegans F37C12.2. *Ce*-F37C12.2 is found downstream of F37C12.3 in the same operonic structure CEOP3416 in C. elegans and *C. briggsae.* The new intron annotated with an asterisk (*) is found in B. malayi and *S. ratti,* but the two introns are separated by nine nucleotides of coding sequence in a protein-driven alignment: the orthology of these introns is thus debatable.

C. elegans operons are characterised by close apposition of the cotranscribed genes, with ~100 bp between the polyadenylation signal of the upstream gene and the acceptor splice site at the 5′ end of the downstream gene. The intergenic distances in putative operons from other nematode species, measured from the end of the 3′ UTR (defined by the site of poly(A) addition) to the SL addition site in cDNAs, range from 89 bp *(O. tipulae)* to 838 bp *(A. suum).* The intergenic distances in the rhabditine (clade V of [37]) and tylenchine (clade IV) nematodes tend to be shorter than those in spirurine (clade III) species, though one rhabditine operon (N. brasiliensis NBOP1624) has a larger intergenic region than the B. malayi operons. In C. elegans, the surveyed pairs of operonic genes have between one and three introns, with an intron size range of 45–336 bp (Figure 2 and Table 2). Other species have different gene structures, displaying both gain and loss of introns compared to C. elegans. Intron sizes in the other nematodes sampled ranged from 39 to 2,308 bases. N. brasiliensis also has longer introns than the other rhabditine species surveyed. For a comprehensive list of intron and intergenic region lengths, see Table S5.

### Characterisation of Transcripts from B. malayi OP1032

**Figure 3:**
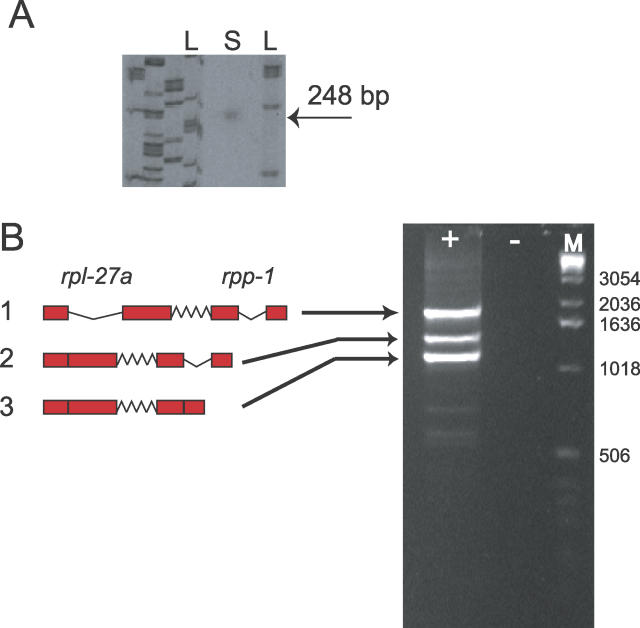
Mapping the 5′ End of *Bm-rpa-1* mRNA and Isolation of Processing Intermediates of the *Bm*-*rpl-27a*/*rpa-1* Polycistronic pre-mRNA (A) An autoradiograph showing the primer extension products from *B. malayi rpa-1* mRNAs. The single observed product is 248 bp. This is consistent with the expected size of an SL1 *trans*-spliced cDNA. L, M13 sequencing ladder; S, primer extension product. (B) The processing intermediates of the *rpl-27a* and *rpa-1* polycistron amplified by RT-PCR. Fragment 1: no processing, introns in both genes present. Fragment 2: processing intermediate with *rpl-27a* intron removed. Fragment 3: processing intermediate with both the *rpl-27a* and the *rpa-1* introns removed. +, reaction with reverse transcriptase added; −, sham reaction with no reverse transcriptase added; M, DNA size markers.

The increased spacing of the genes in the B. malayi and A. suum putative operons compared to C. elegans might indicate that, while these genes are syntenic with their C. elegans orthologues, they are not in an operon. The B. malayi OP1032 *(rpl-27a/rpa-1)* operon was thus examined in detail. Primer extension of the 5′ end of steady-state *Bm-rpa-1* transcripts was performed on specifically reverse-transcribed cDNA. A single extension product was identified. The size of this product was consistent with the *trans-*splicing of a 22-nucleotide leader to the splice acceptor site identified upstream of the initiation AUG codon (Figure 3A). This, in the absence of other evidence, would suggest the use of only a single class of *trans-*SL, 22 bases in length on this downstream gene. Reverse transcription–coupled PCR (RT-PCR) of *Bm-rpa-1* using C. elegans SL2 and SL2-like variant primers in conjunction with a specific *rpa-1* primer failed to yield any products (unpublished data). RT-PCR with SL1 as the 5′ primer was, however, successful.

RT-PCR was also used to investigate the presence of operon-derived pre-mRNAs for the *Bm-rpl-27a/rpa-1* gene pair. Using primers specific to the 5′ end of *rpl-27a* and the 3′ end of *rpa-1,* three major pre-mRNA intermediates were amplified (Figure 3B). Sequencing of these products revealed that the largest corresponded to a dicistronic transcript containing the *cis-*spliced introns of both genes and the intergenic spacer. The other two corresponded to a product wherein the intron in *rpl-27a* had been *cis-*spliced, and a product wherein the introns in both genes had been *cis*-spliced. No product corresponding to a dicistronic, partially processed pre-mRNA with only the *rpa-1* intron spliced was identified.

### Identification of Variant SL Sequences at the 5′ End of Operonic Transcripts in Other Nematode Species

The variant SL2-like sequences in C. elegans were first discovered during sequencing of the 5′ ends of cDNAs for an essentially random selection of genes *(pkc-1, ckn-2, tra-2)* before these genes were known to reside in operons [20,23–25]. Unbiased isolation of 5′-complete cDNAs was achieved through the use of 5′ TMG cap-dependent cDNA amplification. For each gene, several (between nine and 126) clones were sequenced to identify the SLs present. The sequences found at the 5′ ends of four mRNAs (the upstream genes *rpa-0* and *rpl-27a,* and the downstream genes *tct-1* and *rpa-1*) were examined in four species *(A. suum, B. malayi, N. brasiliensis,* and *S. ratti).* In P. pacificus only the 5′ end of the downstream gene *rpa-1* was examined. In *S. ratti,* an additional gene, whose orthologue in C. elegans (Y82E9BR.3) is not operonic, was also surveyed. In *A. suum* and *B. malayi,* for all genes examined, the SLs identified were clearly SL1-like, with the vast majority (98%) identical to the canonical SL1 (Table 3). This concurs with a previous analysis of the 5′ SL sequences found on a random selection of A. suum embryo mRNAs, all of which were SL1 [19]. Five variant SL1 sequences were also identified in these two species (see below). In S. ratti the SL sequences found at the 5′ end of all genes were all SL1-like variants (see below). The majority (>95%) of *P. pacificus rpa-1* products contained SL2-like SLs, but SL1-containing clones were also isolated. In *N. brasiliensis,* the canonical SL1 sequence was identified at the 5′ end of *Nb-rpl-27a* and *Nb-rpa-0* transcripts, while both SL1 and SL2-like SLs were identified on *Nb-rpa-1* and *Nb-tct-1* (Table 3). However, unlike P. pacificus and *C. elegans,* SL1 was frequently *trans-*spliced to the 5′ ends of the putative downstream genes *Nb-rpa-1* and *Nb-tct-1* (31% and 85% of sequenced clones, respectively).

**Table 3:**
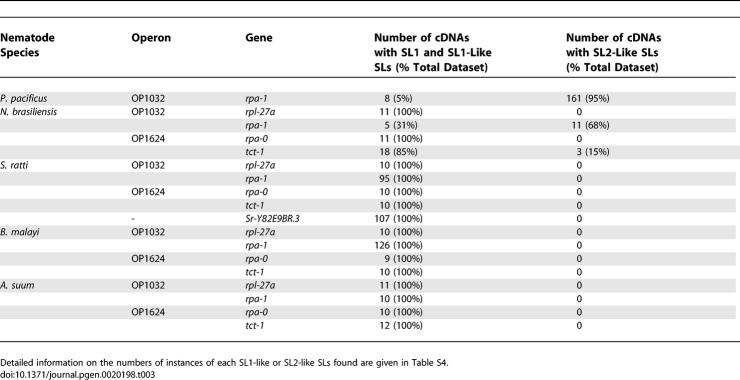
SL Usage in Downstream Genes Found in Operons in Different Nematode Species

### Diversity of SL2-Like Sequences

**Figure 4:**
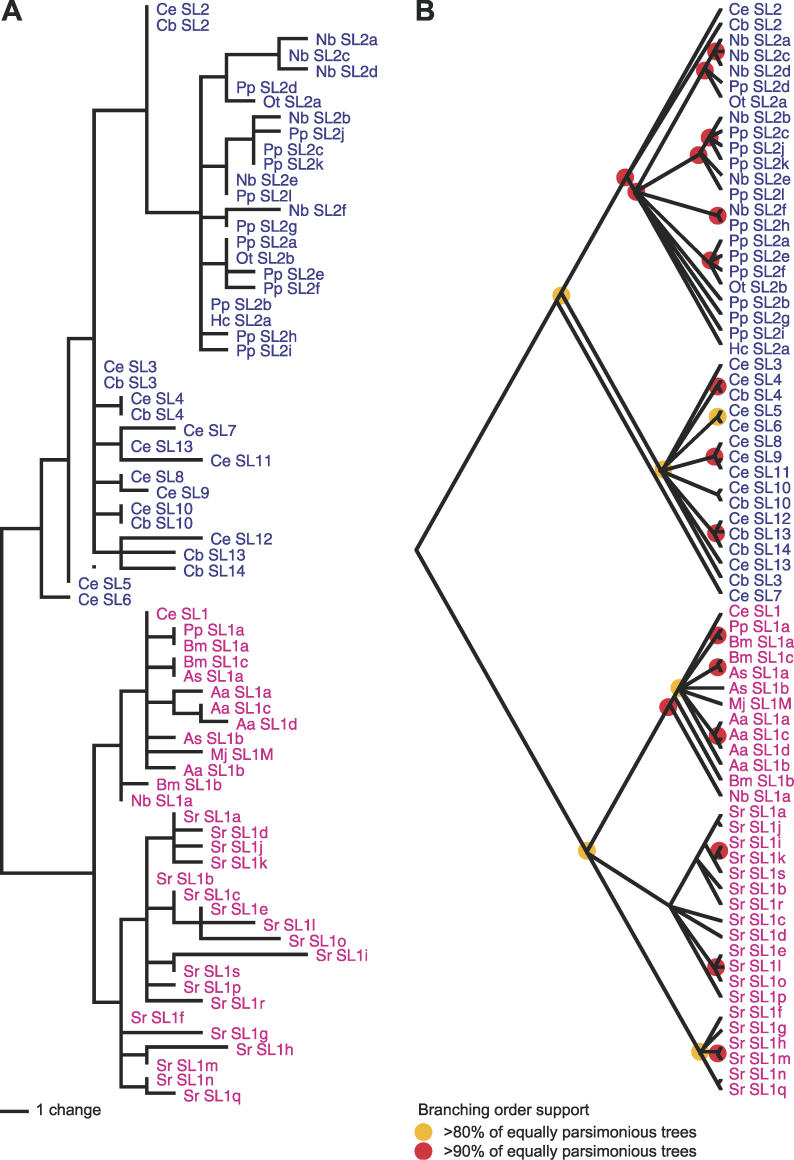
The Evolution of Nematode SLs (A) Consensus maximum parsimony phylogram of SL relationships. (B) Majority-rule cladogram indicating percentage representation of nodes in 10,000 trees of the same optimal length. Nodes with less than 50% support are collapsed as polytomies.

In P. pacificus and *N. brasiliensis,* 17 different SL2-like sequences were identified *trans*-spliced to downstream genes. None were identical to any C. elegans SL2 sequence, and seven were present in more than one clone. These sequences are classified as SL2-like based on the spacing of the 5′ GGTWW motif from the central CCCA motif (three or four bases in C. elegans SL2-like sequences, but five bases in SL1), the spacing of the central motif from the 3′ AG (seven or eight bases in C. elegans SL2-like sequences, but six bases in SL1), and the identity of the last three bases (AAG in all C. elegans SL2-like sequences save one, *Ce*-SL7, but GAG in SL1). SL2-like sequences previously identified in O. tipulae [32] and H. contortus [34] also fit this model. Identical SL2-like sequences were shared by multiple species (Table 4): N. brasiliensis *Nb*-SL2e was identical to P. pacificus *Pp*-SL2l; *P. pacificus Pp*-SL2b was identical to the H. contortus SL2-like sequence; and *Pp*-SL2a was identical to *O. tipulae Ot-*SL2b. Thus, while there appears to be a core group of conserved SL2-like sequences, they are not present in all rhabditines. Phylogenetic analysis of SL2-like sequences suggests an independent radiation of variants in each lineage tested (Figure 4). A full list of SL2-like sequences isolated from each species and their relative abundances can be found in Table S4.

**Table 4:**
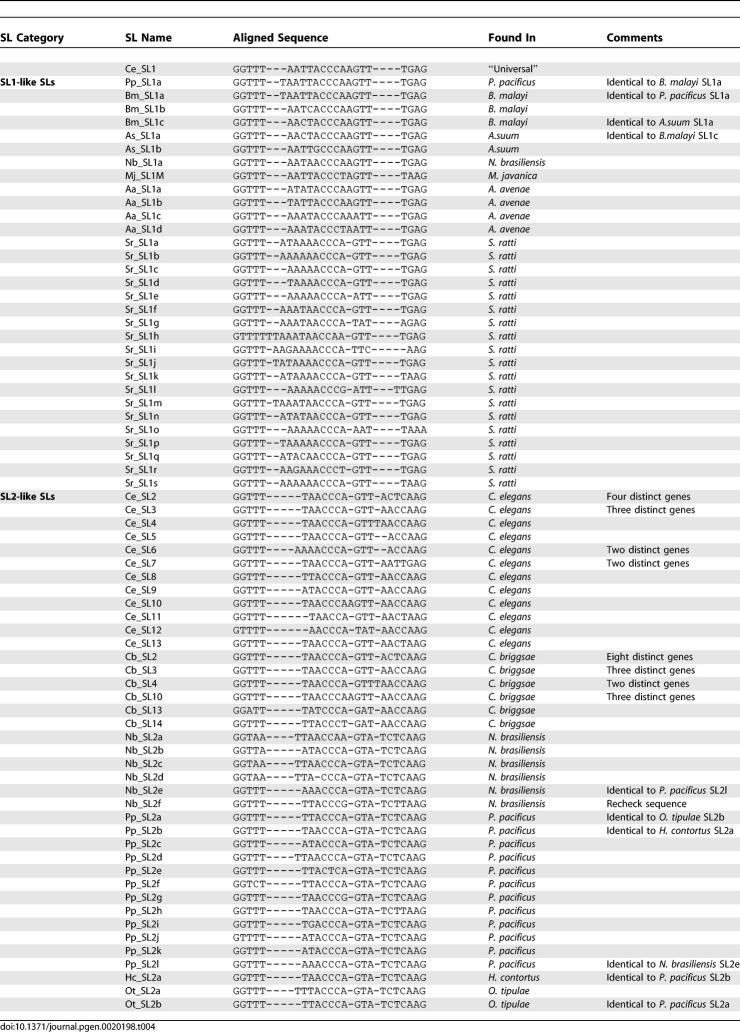
Nematode SL Sequences

### S. ratti Utilise SL1-Like SLs Exclusively

In S. ratti all of the sequences found at the 5′ end of RACE (rapid amplification of cDNA ends) products were identified as SL1 variants. Nineteen different SL1-like sequences were found, with a spacing of five to eight bases between the 5′ GGTTT and the central CCCA, five to six bases between the CCCA and the 3′ AG, and all but four with the last three bases GAG. Parsimony analysis of the sequences indicates that they form a distinct group with the other SL1-like sequences. However, because of the small amount of variable sequence available, the large polytomy within this group was largely unresolved (Figure 4). One S. ratti SL1-like leader sequence has AAA as the last three bases, and is the only SL sequence not to have a 3′ terminal G; this may be a PCR error, but was observed twice. Four of the SL-1 like sequences were found independently three or more times. A full list of SL1-like sequences isolated from S. ratti and their relative abundances can be found in Table S4.

These SL1 variants are similar, but not identical, to those recently identified in another tylenchine nematode, *A. avenae,* where they were found at the 5′ ends of transcripts of the trehalose-6-phosphate synthase genes *Av-tps-1* and *Av-tps-2* [35]. We note that the other described variant SL, SL1M from *M. javanica,* also a tylenchine (clade IV) nematode, was identified using a truncated SL1-derived primer in a RT-PCR screen [43]. The use of the truncated primer will have precluded the discovery of differences in the SL1 sequence upstream of the 3′ terminal SL1M-defining AAG sequence.

Identical variant SL1 sequences, with a single additional T following the conserved 5′ GGTTT motif, were identified at the 5′ end of *rpa-1* in P. pacificus (one instance) and B. malayi (two instances in 125 clones sequenced). Five additional variant SL1, with single substitutions compared to canonical SL1, were identified in *A. suum, B. malayi,* and *N. brasiliensis* (one instance each; see Table S4). One of these variants was identified in both A. suum and B. malayi (*Bm*-SL1c and *As*-SL1a). None of the C. elegans genomic copies of the SL1 gene encode such variant SL1-like SLs. Comparison of these SL1 variants to the SL1 gene sequences contained in the B. malayi WGS sequence failed to identify any cognate genes. However, other variant SL1 genes associated with the 5S sequences were easily identified, indicating that unlike *C. elegans, B. malayi* has polymorphism within the SL1 gene cluster (unpublished data).

## Discussion

Operons are a striking feature of nematode molecular biology, and are absent from other well-studied models such as arthropods and mammals. Outside of the nematodes only two other metazoans, the urochordates Oikopleura dioica and *Ciona intestinalis,* have thus far been found to have operons as a common genomic feature [6,7]. More than 20% of the transcriptome of C. elegans is organised as operons [28]. That these operons are relatively stable during evolution is illustrated by the finding that 97% are conserved in *C. briggsae,* which last shared a common ancestor with C. elegans about 100 million years ago (MYa) [31]. While the bulk of local synteny between these two species has been broken through an extraordinarily high rate of mainly intrachromosomal rearrangement, operons have been relatively protected from these events [44]. As the downstream gene(s) in an operon have lost independent promoter elements, it is likely that there is a selection against break-up of operons, once formed, as isolated downstream genes will be promoterless and nonfunctional. Thus, given an operonic mode of transcription, a prediction is that through evolutionary time an ever-larger number of genes will become operonic, and that distinct lineages may have distinct sets of operonic genes. There is likely to be a limit to the level of operonisation a transcriptome can undergo, as many genes need to be independently and rapidly regulated, and their incorporation into an operon may affect their, or their partners', function. The incorporation of genes into operons requires minimally that the downstream genes have transcriptional profiles compatible with the promoter-donating upstream partners. The rate at which operonisation will take place is unknown, but the relative stability of the proportion and composition of operonic genes in C. elegans and C. briggsae (~3% change in ~100 MYa; [30]) suggests that stability was achieved before these taxa last shared a common ancestor. So when in nematode evolution did operons arise?

Operons were known to exist in another rhabditomorph species, *O. tipulae,* relatively closely related to *Caenorhabditis* [32], and have been identified in the satellite model nematode P. pacificus [33] (a diplogasteromorph; Diplogasteromorpha are a sister clade to the Rhabditomorpha within Rhabditina; see Figure 1 and [38]). We have identified conserved operonic structures in an additional, parasitic rhabditomorph *(N. brasiliensis),* the animal-parasitic tylenchine *S. ratti,* and in the spirurines A. suum and *B. malayi.* These conserved operons were also found in *P. pacificus.* The presence of conserved operons in three major nematode clades implies that these operons formed, and were functional, in the last common ancestor of the Rhabditida (see Figure 1). In the absence of informative fossils, dating this ancestor must rely on molecular dating techniques, which are known to have particular problems in Nematoda due to increased rates of molecular evolution [45]. However, the nematode myoglobin genes behave consistently in phylogenetic analyses, showing a relatively constant rate of molecular change, and concur with multigene analyses in placing C. elegans and C. briggsae about 105 MYa apart (unpublished data) [31]. The separation of the Spirurina *(A. suum* and *B. malayi)* and Rhabditina *(C. elegans, N. brasiliensis,* and *P. pacificus)* is estimated at >500 MYa using myoglobin data [45]. These operons may thus have been present in nematodes since the Silurian era. One operon, the orthologue of C. elegans CEOP5428 *(fib-1/rps-16)* was absent from B. malayi but present in C. briggsae and *P. pacificus.* CEOP5428 is a candidate operon gain event in the lineage leading to the Rhabditina, and may be a discriminant molecular synapomorphy for this major clade. The soon-to-be-completed genome sequence of B. malayi will act as a very useful source of information concerning the evolution of operon structures [46]. Additional nematode genomes are being sequenced. While most of these are additional rhabditines (including three *Caenorhabditis* species, *Heterorhabditis bacteriophora, Haemonchus contortus, Ancylostoma caninum,* and P. pacificus), the list includes the dorylaim *Trichinella spiralis,* an outgroup to the Rhabditida*,* and also the tylenchomorph Meloidogyne hapla and the ascaridomorph *A. suum.* With these genomes assembled, the pattern of operon gain (and loss) will be come clearer, and models for operon evolution better parameterised.

Our analysis of SL2 usage in operonic genes revealed several startling findings. SL2- like sequences appear to be confined to the rhabditine group. Within this group they are specifically associated with the resolution of polycistronic transcripts, although there is one report of SL2-like sequences being *trans*-spliced to an H. contortus gene that may not be in an operon [31]. In our survey we found SL2-like sequences *trans*-spliced exclusively to the downstream genes in operons. SL1 is also used for *trans*-splicing to downstream operonic genes *in P. pacificus* and *N. brasiliensis.* In P. pacificus and *C. elegans,* SL1 addition to downstream genes is rare. However, in *N. brasiliensis,* SL1 was a dominant species (31%–85%). This indicates that the mechanisms in C. elegans (and presumably P. pacificus) that selectively utilise SL2 to resolve downstream genes in operonic transcripts may behave differently in *N. brasiliensis.*

C. briggsae has 18 defined SL2-like SL snRNA genes (Tom Blumenthal, personal communication) [31]. These have six different SL segments, only one of which is distinct from those found in *C. elegans.* Analysis of the SL2 snRNA genes suggests independent radiation of these genes in each species from a smaller pool of ancestral SL2-like snRNA genes (unpublished data). Our analysis of the other rhabditine datasets indicates that SL2-like gene families may be behaving in a similar way in other nematodes. In O. tipulae and H. contortus a few SL2-like snRNA genes have been identified. These carry SL2-like SLs not found in *Caenorhabditis* species, and our survey of P. pacificus and N. brasiliensis operons identified many new SL2-like SLs. On the downstream gene in the OP1032 operon we found 17 different SL2-like SLs. However, each species preferentially utilised a single SL: *Pp*-SL2a in P. pacificus (62% of all SL2 sequences isolated) and *Nb-*SL2a in N. brasiliensis (57% of all SL2 sequences isolated). Within each species' dataset, the other SL2-like sequences are related to the dominant SL2-like sequences identified (see Figure 4).

A number of the SL2-like SLs isolated from both species were found only once, and thus could be artefacts generated in the oligo-cap cloning procedure. The others were identified from two to 106 times each. The most common SL2-like SL in P. pacificus is identical to the SL2-like SL identified in *H. contortus,* and the third most abundant is identical to one of the SL2-like SLs from *O. tipulae.* While phylogenetic analysis of these short sequences is compromised by their high identity and limited length, the N. brasiliensis and P. pacificus SL2-like SLs cluster with the O. tipulae and H. contortus sequences, and are distinct from those of the *Caenorhabditis* species. This pattern affirms and extends the *C. elegans–C. briggsae* model of independent radiation and diversification of SL2-like SLs in different rhabditine lineages.

In contrast to the situation in rhabditines, in the spirurines A. suum and B. malayi the putative downstream genes in operons received SL1. SL2-like SLs were not observed in our analyses, and they were not identified in a survey of full-length cDNAs from ~200 randomly selected embryonically expressed genes [19]. The intergenic spacing of the genes in B. malayi and A. suum operons was larger than that observed in most other species. It has previously been noted that intons in B. malayi genes are on average larger (and more numerous) than in C. elegans [47], and this proportional increase in nontranslated DNA may extend to intergenic distances in polycistrons. The spacing of the genes might suggest they are non-operonic, but we were able to demonstrate polycistronic pre-mRNAs and splicing intermediates for B. malayi OP1032. While we cannot exclude the possibility these splicing intermediates represent dead-end products, these data indicate that polycistronic pre-mRNA can be easily isolated, and studies in other organisms such as Oikopleura dioica have identified similar splicing intermediates [6]. Thus, while operons are present in A. suum and *B. malayi,* they are not resolved by *trans*-splicing with SL2-like SLs, implying that these two molecular phenomena have independent evolutionary origins.

Several variant SL1-like sequences were identified in *A. suum, B. malayi,* and *P. pacificus.* The C. elegans SL1 gene family is present in a monomorphic tandem array of ~110 copies with the 5S rRNA gene. While we were unable to identify the *B. malayi* variant SL1 sequences in the B. malayi whole genome shotgun assembly, we did identify a number of other variant SL1 genes. The assembly procedure may have conflated SL1-like SLs in the 5S rRNA gene repeat.

Variant SL1-like SLs have been observed twice previously, both in nematodes of the Tylenchina. In the tylenchomorph M. javanica a SL1 with a single base change (SL1M) was noted in full-length cDNAs, and when tested was found to be differentially used compared to the canonical SL1 [36]. In the aphelenchomorph *A. avenae,* four different SL1-like SLs were identified at the 5′ ends of trehalose phosphate synthase *(tps)* genes [35]. We surveyed SL usage in another tylenchine nematode, the panagrolaimomorph *S. ratti.* Here we found a wide diversity of SL1-like SLs being used at the 5′ end of downstream genes. None was identical to the SL1-like SLs previously identified in M. javanica and *A. avenae.* A survey of three other S. ratti genes, two of which are the upstream genes in operons, and a third not in an operon in *C. elegans,* yielded an overlapping set of 19 SL1-like sequences. This data suggests that a family of SL1-like SLs may be utilised for *trans-*splicing operonic and non-operonic genes. Importantly, canonical SL1 was not observed at the 5′ end of any clones in the S. ratti dataset. Previous surveys of SL1 presence and use in tylenchine nematodes, including *S. ratti,* have used the canonical SL1 sequence as a probe or PCR primer. As the SL1-like sequences are similar, particularly at the 3′ end, a canonical SL1 primer will have been able to promiscuously amplify from many of the SL1-like SLs. Thus, it may be that canonical SL1 is lacking in some of these species.

The Nematoda can be divided into three subclasses: Chromadoria, Enoplia, and Dorylaimia [38]. All the species we have studied are members of Chromadoria. *Trans*-splicing and SLs are likely to have been present in the last common ancestor of the phylum, as *trans*-splicing has been observed in the dorylaimian T. spiralis [14,48]. We note, however, that the presence of SL1 was inferred in T. spiralis through PCR amplification using an SL1 primer: the sequence of T. spiralis SLs has not been independently confirmed. We have not been able to identify canonical SL1 at the 5′ end of T. spiralis ESTs derived from cDNA libraries enriched for full-length transcripts, but have evidence of non–SL1-like *trans*-splicing (unpublished data).

Mapping these findings onto the nematode phylogeny, we can propose the following outline of the evolution of *trans*-splicing, families of *trans-*SLs, and operons (assuming, parsimoniously, a minimum of events of gain of these complex features; Figure 1). SL2-like SLs arose in and are confined to the Rhabditina, the group that includes *C. elegans,* where they are intimately associated with polycistronic pre-mRNA resolution. However, operons probably arose earlier in nematode phylogeny, and in other rhabditid suborders (Tylenchina and Spirurina) operonic transcripts are resolved by *trans*-splicing to SL1 or SL1-like SLs. In Spirurina, variant SLs are very similar to SL1 in sequence, and rare: most operonic transcripts are resolved using canonical SL1. In contrast, in Tylenchina, the SL1-like SLs are more distinct in sequence from canonical SL1, and SL1 itself may be excluded from resolution of operonic pre-mRNAs or absent entirely from these species. Thus, operons in different nematode clades are associated with very different sorts of SLs, arguing for independent divergence from a common ancestor, where, we suggest, operons will have been resolved by SL1 *trans*-splicing. Preliminary analysis of the B. malayi whole-genome shotgun suggests that only a small set of operons are conserved between B. malayi and *C. elegans,* and the majority of predicted B. malayi operons appear to be novel gene aggregations (E. Ghedin and D. Spiro, personal communication). Why might novel SLs have arisen for operonic resolution in rhabditine nematodes? We speculate that as operons became a major mode of transcriptional organisation, the need for special mechanisms to resolve them became necessary. *Trans*-splicing with SL1 does not normally happen within the core of a pre-mRNA (although it can), and efficient linkage of polyadenylation to downstream *trans*-splicing may have been achieved by evolution of novel (SL2-like) SL genes with particular interactions with the splicing and polyadenylation machineries.

The essential differences observed in SL recruitment to operon-resolving RNA-processing complexes, SL2-like and standard SL1 *trans*-splicing in C. elegans is conferred by the non-SL portion of the SL snRNAs, which differ in length and sequence content. To further clarify the origins of the phenomenon of operonic gene organisation in the Nematoda it will be necessary to examine the SL snRNA genes from a range of species. The emerging nematode genome sequences will supply much of the data required for these analyses.

## Methods

### Identification of potential operon partners.

B. malayi (strain TRS, from TRS Labs, Athens, Georgia, United States) was selected as the initial test organism because of the availability of large amounts of genomic sequence generated by the Filarial Genome Project [49] and its phylogenetic distance from C. elegans [37]. A targeted cloning by synteny approach was taken to isolate conserved operonic structures from *B. malayi,* as sequencing of over 180 kb of B. malayi genomic DNA contigs had not yielded any candidate operonic gene pairs, despite high gene density [47,50]. The bias in C. elegans operonic genes towards ribosomal proteins [28] and the B. malayi EST dataset [51] was utilised to identify potential operon partners. Representative cDNA clones were obtained from the Filarial Resource Center or from the Blaxter lab archive and sequenced in full. We designed oligonucleotide primer sets to amplify each gene individually, and used these to test their close linkage in genomic DNA by PCR (Table 1). Primer sequences are presented in Table S2.

Additional nematode species were surveyed for genes potentially in operons by identifying potential operon partners in clustered EST data available in NEMBASE (http://www.nematodes.org) [40,52]. DNA and RNA were isolated from N. brasiliensis (maintained in Sprague-Dawley rats, which were obtained from Yvonne Harcus, Institute of Immunology and Infection Research, University of Edinburgh, United Kingdom), P. pacificus (strain PS312 obtained from the *Caenorhabditis* Genetics Center [http://www.cbs.umn.edu/CGC]), S. ratti (homogonic isofemale line ED231 obtained from Mark Viney, School of Biology, University of Bristol, United Kingdom), and A. suum (wild material from Scottish abattoirs obtained from Malcolm Kennedy, Institute of Biomedical and Life Sciences, University of Glasgow, United Kingdom). Nematode material was homogenized in lysis buffer (110 mM NaCl, 110 mM Tris-Cl [pH 8.5], 55 mM EDTA, 1.1% SDS, 1.1% 2-mercaptoethanol) and proteinase K (100 μg/mL; Sigma, http://www.sigma.com) and DNase-free RNAse (100 μg/ml) for 1 h at 65 °C. Genomic DNA was isolated from extracts by phenol-chloroform extraction and precipitation with isopropanol using standard methods. PCR was performed using LongRangeTaq (Stratagene, http://www.stratagene.com) or Expand Long Range Polymerase (Roche, http://www.roche.com) using >300 ng of genomic DNA and 10 pmol each primer (see Table S2). PCR products were cloned in pCR4.0-TA cloning vector (Invitrogen, http://www.invitrogen.com) and transformed into TOP10 cells. The inserts were sequenced using BigDye 3.0 reagents and an ABI 377 sequencer (Applied Biosystems, http://www.appliedbiosystems.com). Introns were mapped by comparison to the clustered EST data for each species available in NEMBASE, or predicted based on similarity to relevant proteins in the public databases.

### Isolation of novel SLs from the 5′ end of cDNAs.

Total RNA was isolated from homogenized nematode material using TRIzol (Invitrogen), and some samples were further purified using the RNeasy mini-purification columns (Qiagen, http://www1.qiagen.com). RNA utilised for the generation of RACE fragments was isolated from B. malayi (mixed adults), N. brasiliensis (mixed adults), P. pacificus (mixed population of all stages), S. ratti (mixed free-living stages), and A. suum (uterine material). 5′ RACE fragments for each gene were isolated for each chosen gene using the GeneRacer Kit (Invitrogen). This method generates full-length cDNAs by utilising the 5′ cap structure to first protect the mature mRNA from modification, and then to permit directed ligation of an RNA oligonucleotide tag sequence to the 5′ end of mRNAs. Briefly, 5 μg of total RNA or 200 ng of poly(A)+ mRNA was calf intestinal phosphatase and TAP treated according to the manufacturer's instructions. The GeneRacer RNA oligo was then ligated on the 5′ end of the treated RNA, and this modified RNA was reverse-transcribed using Superscript II (Invitrogen) and GeneRacer oligo(dT) primer. RACE fragments for each gene were then isolated by PCR using Thermozyme (Invitrogen) or AGS-Gold (Hybaid, http://hybaid.org) Taq polymerases, gene-specific reverse primers (primer sequences available in Table S2) and the GeneRacer 5′ oligo as the forward primer according to manufacturer protocols. Some of the isolated RACE fragments required a second round of amplification using a nested gene-specific reverse primer and the Gene Racer 5′ nested primer. Isolated PCR products were gel purified (Qiagen Gel Purification Kit) and cloned into pCR4.0-TA cloning vector (Invitrogen). For each gene, several recombinant (up to 130) clones were selected and sequenced at the 5′ end using vector primers or gene-specific reverse primers. The SL portion of the sequence was identified by comparison to the cognate genomic DNA sequence.

### Isolation of polycistronic intermediates for the B. malayi orthologue of OP1032, and primer extension mapping of *Bm-rpa-1* mRNAs.

RT-PCR was performed on 5 μg of DNAse I (Stratagene)–treated total RNA from B. malayi using the set of primers designed to isolate the operon structure. PCR was performed using AGS-Gold Taq (Hybaid) using standard reaction conditions and 35 cycles of amplification. The PCR primers from within each gene served as positive controls, while a reverse transcriptase–negative reaction was used to control for contaminating genomic DNA. Products from PCR primer pairs spanning the operon were cloned and sequenced to identify their content.

To map the 5′ end of the downstream gene *Bm-rpa-1,* a primer extension reaction was carried out using a γP^32^-radiolabelled reverse primer directed against the first exon of *Bm-rpa-1.* RT was carried out using 10 μg of total RNA, the radiolabeled primer, and AMV reverse transcriptase (Sigma). Products were analysed on 4.5% polyacrylamide gel along with an M13 sequencing ladder as a size marker and autoradiographed.

### Phylogenetic analysis of SL1, SL2, and variant SLs.

SL1, SL1-like, and SL2-like SLs were aligned by eye (Table 4). Analysis was carried out in PAUP v4b10, using maximum parsimony. Due to the short, largely conserved sequences, both neighbour-joining and Bayesian analyses were uninformative. Ten independent runs of maximum parsimony analysis were carried out with a random starting tree, and 1,000 best trees were kept from each replicate. Insertion–deletion characters (gaps) were treated as a “fifth base.” The majority-rule consensus of these 10,000 trees (retaining groups with >50% representation) was calculated.

## Supporting Information

Table S1 The C. elegans SL2-Like Gene Family (49 KB DOC)

Table S2 Primers Used in This Study (161 KB DOC)

Table S3 Cytoplasmic Ribosomal Protein Genes of B. malayi
(156 KB DOC)

Table S4 Alternate SL Usage Identified in *A. suum, B. malayi, N. brasiliensis, S. ratti,* and *P. pacificus*
(158 KB DOC)

Table S5 Conserved Operons in Species Other than C. elegans
(75 KB DOC)

### Accession Numbers

Sequences reported in this manuscript have been submitted to EMBL (http://www.ebi.ac.uk)/GenBank (http://www.ncbi.nlm.nih.gov)/DDBJ (http://www.ddbj.nig.ac.jp) under the accession numbers DQ516385–DQ516398 and DQ993362–DQ993521.

## Abbreviations

EST: expressed sequence tag
MYa: million years ago
RACE: rapid amplification of cDNA ends
SL: spliced leader
snRNA: small nuclear RNA
TMG: trimethylguanosine
UTR: untranslated region
