# Supplementary material for: Operon Conservation and the Evolution of trans-Splicing in the Phylum Nematoda
Source: PLoS Genet. 2006 Nov 24;2(11):e198. doi: 10.1371/journal.pgen.0020198 (PMC1657053; doi:10.1371/journal.pgen.0020198)
Supplement: Table S1 — (49 KB DOC) [file pgen.0020198.st001.doc]

**Supplementary Materials, Table S1: The *C. elegans* spliced leader 2-like gene family**

| WB Gene ID ^1^ | Sequence Name ^2^ | CGC Name ^3^ | SL name ^4^ |
| --- | --- | --- | --- |
| WBGene00004833 | ZK1248.18 | sls-2.1 | SL2-1 |
| WBGene00004834 | C17C3.16 | sls-2.2 | SL2-2 |
| WBGene00004835 | C17C3.17 | sls-2.3 | SL2-3 |
| WBGene00004836 | R12E2.16 | sls-2.4 | SL2-4 |
| WBGene00004837 | B0280.14 | sls-2.5 | SL3-1 |
| WBGene00004838 | W06D4.7 | sls-2.6 | SL3-2 |
| WBGene00004839 | W06D4.8 | sls-2.7 | SL3-3 |
| WBGene00004840 | Y75B8A.38 | sls-2.8 | SL4 |
| WBGene00004841 | M01E5.7 | sls-2.9 | SL5 |
| WBGene00004842 | F23B2.14 | sls-2.10 | SL6-1 |
| WBGene00004843 | F23B2.15 | sls-2.11 | SL6-2 |
| WBGene00004844 | F36H12.19 | sls-2.12 | SL7-1 |
| WBGene00004845 | R13H9.7 | sls-2.13 | SL7-2 |
| WBGene00004846 | W09D6.7 | sls-2.14 | SL8 |
| WBGene00004847 | W09D6.8 | sls-2.15 | SL9 |
| WBGene00004848 | B0280.15 | sls-2.16 | SL10 |
| WBGene00004849 | ZK354.12 | sls-2.17 | SL11 |
| WBGene00004850 | B0280.16 | sls-2.18 | SL12 |
| WBGene00004851 | F36H12.20 | sls-2.19 | SL13 |

1 The WormBase unique gene identifier.

2 The “cosmid.gene” version of the gene name.

3 The *Caenorhabditis* Genetics Center approved gene name.

4 SL names as designated by Prof. Tom Blumenthal in WormBase.
