# Supplementary material for: Operon Conservation and the Evolution of trans-Splicing in the Phylum Nematoda
Source: PLoS Genet. 2006 Nov 24;2(11):e198. doi: 10.1371/journal.pgen.0020198 (PMC1657053; doi:10.1371/journal.pgen.0020198)
Supplement: Table S2 — (161 KB DOC) [file pgen.0020198.st002.doc]

**Supplementary Materials, Table S2: Primers used in this study**

**Supplementary Materials, Table S2a:** Primers used to isolate operons and 5’ RACE fragments, *B. malayi*

| **Identification of Operons in *B.malayi*, 5’ RACE of selected transcripts, primer extension of *Bm-rpa-1* and RT-PCR of intermediate splice products of BMOP1032** | | |
| --- | --- | --- |
| Bm-rpL27a.F1 | GGCTACATCAAAGAAAAAGACAAGG | Long range PCR and 5’RACE of *Bm-rpl27a* forward primer |
| Bm-rpL27a.R1 | GCAACCAATACACAGGCACCAC | Long range PCR and 5’RACE of *Bm-rpl27a* reverse primer |
| Bm-rpL27a.R2 | CCTTCTCCGATATCAGTGACCACAGC | 5’RACE of *Bm-rpl27a* nested reverse primer |
| Bm-roPl.FI | ATGGCAAACCAAGAATTAGC | Long range PCR and 5’RACE of *Bm-rpa-1* forward primer |
| Bm-rpPI.RI | CAACTCCTTCCAAGGCCTTAGC | Long range PCR and 5’RACE of *Bm-rpa-1* reverse primer |
| Bm-rpPI.R4 | ACCACTACCAACACTTGAACT | 5’RACE of *Bm-rpa-1* nested reverse primer and primer extension |
| Bm-rpPO.FI | TACGGTCTTGTTGTGCGGCAGG | Long range PCR *Bm-rpa-0* forward primer |
| Bm-rpPO.RI | CTTAATCGAAAAGTCCGAATCC | Long range PCR *Bm-rpa-0* reverse primer |
| Bm-rpp0.F3 | GAAGACAAAGCAACGTGGAAAG | 5’RACE of *Bm-rpa-0* forward primer |
| Bm-rpp0.R2 | GCCTTAGCAGGAGCACCACGTCG | 5’RACE of *Bm-rpa-0* reverse primer |
| Bm-rpp0.R3 | GAAGCTCCCACTTTCTCGCCAGTC | 5’RACE of *Bm-rpa-0* nested reverse primer |
| Bm-tph-1.F1 | CGATATGTTGATCTTCAAGGATG | Long range PCR and 5’RACE of *Bm-tct-1* forward primer |
| Bm-tph-1.RI | TTGTTTTTCTTCAATGAGTGCCTCCTT | Long range PCR *Bm-tct-1* reverse primer |
| Bm-tph-1.R2 | GAGTGCCTCCTTCACAAGCATC | 5’RACE of *Bm-tct-1* reverse primer |
| Bm-tph-1.R3 | CTTGTAGCCGATGGTTGAGCAC | 5’RACE of *Bm-tct-1* nested reverse primer |
| Bm-FIB-l.FI | TGCTATAAATGCCCACCATTTC | Long range PCR *Bm-fib-1* forward primer |
| Bm-FIB-I.RI | CAACGGACGGTACTCAGCGACC | Long range PCR *Bm-fib-1* reverse primer |
| Bm-rpSI6.FI | GTTGTTACACAATCTGTGCAGG | Long range PCR *Bm-rps-16* forward primer |
| Bm-rpSI6.RI | CAAATTACGATCATACGATACC | Long range PCR *Bm-rps-16* reverse primer |
| Bm-rpl26.F1 | CGAGGACATTATAAAGGAAACG | Long range PCR *Bm-rpl-26* forward primer |
| Bm-rpl26.R1 | GTATGTTTACCTTCAACAGGCC | Long range PCR *Bm-rpl-26* reverse primer |
| Bm-suf-1.FI | TGAGTTCGCTTTCGATTGTAAG | Long range PCR *Bm-suf-1* forward primer |
| Bm-suf-I.RI | GTTTCACAGTATTCCCATGTCT | Long range PCR *Bm-suf-1* reverse primer |
| Bm-rpL5.FI | GTAGGATGGGATTCGTGAAGG | Long range PCR *Bm-rpl-5* forward primer |
| Bm-rpL5.RI | AACCAGTGGCATAAGCTGATGC | Long range PCR *Bm-rpl-5* reverse primer |
| Bm-F54C9.6.FI | ACTGAACCATTTGTCGAACCA | Long range PCR *Bm-F54C9.6* forward primer |
| Bm-F54C9.6.RI | TTGCCGAGTTTGACGTGCGCTG | Long range PCR *Bm-F54C9.6* reverse primer |
| Bm-rpL10.FI | CGACGACCAGCAAGATGCTAC | Long range PCR *Bm-rpl-10* forward primer |
| Bm-rpL10.RI | CAACTCGTGCTACTAAGCCCTG | Long range PCR *Bm-rpl-10* reverse primer |
| Bm-F10B5.2.FI | GCTTGACTCATCATGATTAAG | Long range PCR *Bm-F10B5.2* forward primer |
| Bm-F10B5.2.RI | ACGTGGTGCCGTTTTGACGCTG | Long range PCR *Bm-F10B5.2* reverse primer |
| Bm-rpL36.FI | GGTCGCAGTTGAAGCAGTTGC | Long range PCR *Bm-rpl-36*forward primer |
| Bm-rpL36.RI | CGCTTTATAATCACTTATGAT | Long range PCR *Bm-rpl-36* reverse primer |
| Bm-F37CI2.3.FI | GGAACAAGAATTATACAAGATATG | Long range PCR *Bm-F37C12.3* forward primer |
| Bm-F37CI2.3.RI | CTCACTTTCCAGTTCTGAGATCTC | Long range PCR *Bm-F37C12.3* reverse primer |
| Bm-rpSI2.F2 | CAACAGCAGCTGCAGCAGACG | Long range PCR *Bm-rps-12* forward primer |
| Bm-rpS 12.R2 | TGCATAGCCCAATCCATTCAC | Long range PCR *Bm-rps-12* reverse primer |
| Bm-F54E7.l.FI | CTTACAAATAGCAACATTTACG | Long range PCR *Bm-F54E7.1* forward primer |
| Bm-F54E7.l.RI | AATAGAATACGAATACAGAATC | Long range PCR *Bm-F54E7.1* reverse primer |
| Bm-rpS25.FI | AAAACATCAGCGAAAGCTGGG | Long range PCR *Bm-rps-25* forward primer |
| Bm-rpS25.RI | CCTTCGTGCATCTTGTGTAGAC | Long range PCR *Bm-rps-25* reverse primer |
| Bm-K02B2.4.FI | CCACCGAAAGTCTTCGTCGAAG | Long range PCR *Bm-K02B2.4* forward primer |
| Bm-K02B2.4.RI | TTCACTCGTTCATGGAATCTT | Long range PCR *Bm- K02B2.4* reverse primer |
| Bm-mrpS2.FI | CCTATTCATTACGACAGAAAGG | Long range PCR *Bm-mrs-2* forward primer |
| Bm-mrpS2.RI | GTCATCATTTCCGGGAATCGG | Long range PCR *Bm-mrs-2* reverse primer |
| Bm-mrpL4.FI | ATGTCATCAGCGCAGTTGGTTC | Long range PCR *Bm-mrl-4* forward primer |
| Bm-mrpL4.RI | GAATATATCTGGATGTAAACTG | Long range PCR *Bm-mrl-4* reverse primer |

**Supplementary Materials, Table S2b:** Primers used to isolate operons and 5’ RACE fragments, *A. suum*

| **Identification of Operons in *A. suum* and 5’ RACE of selected transcripts** | | |
| --- | --- | --- |
| As_rpl_27a.F | GTGTCGCATGGTCATGGCAGAATC | Long range PCR *As-rpl27a* forward primer |
| As_rpl_27a.R | GACTCCCTTGATCTTTTGCTCG | Long range PCR *As-rpl27a* reverse primer |
| As_rpl_27a.F1 | GTGTCGCATGGTCATGGCAGAATC | 5’RACE of *As-rpl27a* forward primer |
| As_rpl_27a.R1 | GACTCCCTTGATCTTTTGCTCG | 5’RACE of *As-rpl27a* reverse primer |
| As_rpl_27a.R2 | CGGCCTTGACAACATCAATCACGG | 5’RACE of *As-rpl27a* nested reverse primer |
| As_rpa_1.F | CATCAGGCGGCTACAAACAGCG | Long range PCR *As-rpa-1* forward primer |
| As_rpa_1.R | GTCGAAAAGTCCAAATCCCATG | Long range PCR *As-rpa-1* reverse primer |
| As_rpa_1.F1 | CATCAGGCGGCTACAAACAGCG | 5’RACE of *As-rpa-1* forward primer |
| As_rpa_1.R1 | GTCGAAAAGTCCAAATCCCATG | 5’RACE of *As-rpa-1* reverse primer |
| As_rpa_1.R2 | TGCCGGTGCTGGACCTCCACTG | 5’RACE of *As-rpa-1* nested reverse primer |
| As-rpa-0.F1 | GACGTCGTTCTTTCAAGCTCTA | Long range PCR *As-rpa-0* forward primer |
| As-rpa-0.R1 | GGTCAACATCAGCCTCAATGGC | Long range PCR *As-rpa-0* reverse primer |
| As_rpa_0.F2 | CAGGATGGGCAGGGAAGACAAAG | 5’RACE of *As-rpa-0* forward primer |
| As_rpa_0.R2 | CCAACCACATAAGGAAGCAACTTC | 5’RACE of *As-rpa-0* nested reverse primer |
| As_rpa_0.R3 | CACGTCACACGGCGCTATTGCTCC | 5’RACE of *As-rpa-0* reverse primer |
| As_tph_1.F1 | GTTCAAGAGTTACATAAAGTCG | Long range PCR *As-tct-1* forward primer |
| As_tph_1.R1 | CTTCTCCTTCGGGCTCATCTCG | Long range PCR and 5’RACE of *As-tct-1* reverse primer |
| As_tph_1.F2 | CGTTTCCGATGAAGTTGGTTGATG | 5’RACE of *As-tct-1* forward primer |
| As_tph_1.R2 | CCGTTCTTAGCCATCAGATCAAC | 5’RACE of *As-tct-1* nested reverse primer |

**Supplementary Materials, Table S2c:** Primers used to isolate operons and 5’ RACE fragments, *N. brasiliensis*

| **Identification of Operons in *N. brasiliensis* and 5’ RACE of selected transcripts** | | |
| --- | --- | --- |
| Nb_rpl27a.F1 | CACGTGTCCCATGGACACGGTC | Long range PCR and 5’ RACE of *Nb-rpl27a* forward primer |
| Nb_rpl27a.R1 | CAACAATCCCTTGCCAAGAACC | Long range PCR and 5’ RACE of *Nb-rpl27a* reverse primer |
| Nb_rpp1.F1 | TGGAGCTAAGGCACTTTGCAAC | Long range PCR and 5’ RACE of *Nb-rpa-1* forward primer |
| Nb_rpp1.R1 | CGATGCTTAGTCGAACAGACCG | Long range PCR and 5’ RACE of *Nb-rpa-1* reverse primer |
| Nb_rpp0.F1 | GGCCATCCGTGGTCATTTGTCG | Long range PCR and 5’ RACE of *Nb-rpa-0* forward primer |
| Nb_rpp0.R1 | CTTCTCAGGTCCCATACCGGTG | Long range PCR and 5’ RACE of *Nb-rpa-0* reverse primer |
| Nb_tph1.F1 | CTCATCGGACTCCTTCCCGATG | Long range PCR and 5’ RACE of *Nb-tct-1* forward primer |
| Nb_tph1.R1 | CCTTGAATCTTCTTCTTGAACG | Long range PCR and 5’ RACE of *Nb-tct-1* reverse primer |

**Supplementary Materials, Table S2d:** Primers used to isolate operons and 5’ RACE fragments, *P. pacificus*

| **Identification of Operons in *P. pacificus* and 5’ RACE of selected transcripts** | | |
| --- | --- | --- |
| Pp-rpL27a.F I | GCTTAGAGGACACGTGTCCCACG | Long range PCR *Pp-rpl27a* forward primer |
| Pp-rpL27 a.RI | GTGGGAGAAGAACTTGGCCTTCAC | Long range PCR *Pp-rpl27a* reverse primer |
| Po-rpp1.FI | GTGAGCTAAGGCTTAAAGCAACG | Long range PCR and 5’RACE of *Pp-rpa-1* forward primer |
| Pp-rpp l.R I | GGCGAAGAGACCAGGCCAGAAGGG | Long range PCR and 5’RACE of *Pp-rpa-1* reverse primer |
| Pp-rppO.FI | ATGGGTCCTGAGAAGACCTCG | Long range PCR *Pp-rpa-0* forward primer |
| Pp-rppO.RI | CGCGATTCCGAGCATGCTCTGG | Long range PCR *Pp-rpa-0* reverse primer |
| Pp-tph-I.F I | ATGCTGATCTACAAGGACGCG | Long range PCR *Pp-tph-1* forward primer |
| Pp- tph-I.R I | CTGCTTCTCAATAATGAGAGC | Long range PCR reverse primer |
| Pp-rpL36.FI | CGCAAGGTTACCAAGCTCGAGG | Long range PCR of *Pp-rpl-36* forward primer |
| Pp-rpL36.RI | TACGTTCTGCATCTCGTCACGC | Long range PCR of *Pp-rpl-36* reverse primer |
| Po-F37CI2.3.FI | CAGCTCACATTCAAGGAGGTTG | Long range PCR of *Pp-F37C12.3* forward primer |
| Pp-F37C12.3.RI | CTCTTCATTCAAACACGTCTTC | Long range PCR of *Pp-F37C12.3* reverse primer |
| Pp-rpS 14.F I | GCACGTAAGGGAAAGGTAAAGG | Long range PCR of *Pp-rps-14* forward primer |
| Pp-rpSI4.RI | AAGAGCGCGGAGAGCAGACTGG | Long range PCR of *Pp-rps-14* reverse primer |
| Pp_rps16.F1 | ATGACTAAGGAACTCATCCAGT | Long range PCR of *Pp-rps-16* forward primer |
| Pp_rps16.R1 | GTAAGACTTCTGGTAGCGAGC | Long range PCR of *Pp-rps-16* reverse primer |
| Pp_fib1.F1 | GCTCTGTATTCGGCAACGACGA | Long range PCR of *Pp-fib-1* forward primer |
| Pp_fib1.R1 | CGACCGAACATCATCCCGATTG | Long range PCR of *Pp-fib-1* reverse primer |

**Supplementary Materials, Table S2e:** Primers used to isolate operons and 5’ RACE fragments, *S. ratti*

| **Identification of Operons in *S. ratti* and 5’ RACE of selected transcripts** | | |
| --- | --- | --- |
| Sr_rpl_27a.F1 | GAGGTCGTGGTAATGCTGGAGG | Long range PCR and 5’RACE of *Sr-rpl27a* forward primer |
| Sr_rpl_27a.R1 | CGATCATTGGTTGACTTGGAAC | Long range PCR and 5’RACE of *Sr-rpl27a* reverse primer |
| Sr_rpl_27a.R2 | TTAACTCCAATCATTTTGGTGCG | 5’RACE of *Sr-rpl27a* nested reverse primer |
| Sr-rpp1.FI | ATGACTTCTACTCAAGAACTCG | Long range PCR and 5’RACE of *Sr-rpa-1* forward primer |
| Sr-rpp1.RI | GCAGATCCAGCTCCAGATCCAAT | Long range PCR and 5’RACE of *Sr-rpa-1* reverse primer |
| Sr_rpa_0.F1 | ATGGTTAGGGAAGACAGAAATG | Long range PCR and 5’RACE of *Sr-rpa-0* forward primer |
| Sr_rpa_0.R1 | GCAATTTGACATCACATGGAGC | Long range PCR and 5’RACE of *Sr-rpa-0* reverse primer |
| Sr_rpa_0.R2 | GTAAAGACGAATCCAACGTTTCC | 5’RACE of *Sr-rpa-0* nested reverse primer |
| Sr_tph_1.F1 | CATGTTGAACGTGGTATTGACTT | Long range PCR and 5’RACE of *Sr-tph-1* forward primer |
| Sr_tph_1.R1 | TTCCTTCAGCCATGTTCTCACC | Long range PCR and 5’RACE of *Sr-tph-1* reverse primer |
| Sr_tph_1.R2 | CCATGTGTTAATCTCATCCTCAG | 5’RACE of *Sr-tph-1* nested reverse primer |
| Sr_rps16.F1 | TTGTCCAACCTGTCCAAGTTT | Long range PCR of *Sr-rps-16* forward primer |
| Sr_rps16.R1 | TTTGATAGAAGGCAACAAGAGC | Long range PCR of *Sr-rps-16* reverse primer |
| Ss_fib1.F1 | GTTCAGATCTCGTTGGACCTG | Long range PCR of *Sr-fib-1* forward primer |
| Ss_fib1.R1 | CTTACTTGTTCCCTGAGTTTG | Long range PCR of *Sr-fib-1* reverse primer |
| Sr-rpL36.FI | GAAAAAGGTGTCCGTGTCACC | Long range PCR of *Sr-rpl-36* forward primer |
| Sr-rpL36.RI | TGGATTTCATCACGCTTCTTC | Long range PCR of *Sr-rpl-36* reverse primer |
| Sr-rpSI4.FI | CCGTAAAGGCAAAGTTCGTGAG | Long range PCR of *Sr-rps-14* forward primer |
| Sr-rpSI4.RI | TTCAACAAACGCCATTTATGA | Long range PCR of *Sr-rps-14* reverse primer |
