# Supplementary material for: Operon Conservation and the Evolution of trans-Splicing in the Phylum Nematoda
Source: PLoS Genet. 2006 Nov 24;2(11):e198. doi: 10.1371/journal.pgen.0020198 (PMC1657053; doi:10.1371/journal.pgen.0020198)
Supplement: Table S3 — (156 KB DOC) [file pgen.0020198.st003.doc]

**Supplementary Materials, Table S3: Cytoplasmic ribosomal protein genes of *Brugia malayi***

Orthologues are identified by their GenBank accession numbers (*H. sapiens, D. melanogaster*), genome project identifiers (*C. elegans, D. melanogaster*) and NEMBASE cluster identifiers (*B. malayi*).

**Supplementary Materials, Table S3a:** Large subunit cytoplasmic ribosomal proteins.

| **Protein name** | ***H. sapiens*** | ***D. melanogaster*** | ***C. elegans*** | **Operonic in *C. elegans*** | ***B. malayi*** |
| --- | --- | --- | --- | --- | --- |
| L1, L4 | P36578 | CG8195 | B0041.5 (I) | No | not found |
| L2, L8 | XP_005130.1 | AAF47659.1 | B0250.1 (V) | No | BMC01536 |
|  | not found | AAF47659.1 | B0250.7 (V) | No | BMC00336 |
| L3 | XP_039345.1 | AAF54609.1 | F13B10.2 (II) | No | BMC00329 |
| L5 | XP_028341.1 | AAG22457.1 | F54C9.5 (II) | Yes | BMC01632 |
| L6 | XP_050941.1 | CG11522 | R151.3 (III) | Yes | BMC00250 |
| L7 | XP_035492.1 | AAF52868.1 | F53G12.10 (I) | Yes | BMC00673 |
| L7a | XP_035105.1 | AAF46169.1 | Y24D9A.4 (IV) | No | BMC00621 |
| L9 | XP_047490.1 | AAF53049.1 | R13A5.8 (III) | No | BMC02343 |
| L10 | XP_018268.1 | AAF45349.1 | K11H12.2 (IV) | No | BMC00151 |
| L10a | XP_017704.1 | CG7283 | Y71F9AL.13A (V) | Yes | BMC00576 |
| L11 | P39026 | AAF57560.1 | F07D10.1 (X) and T22F3.4 (V) | No  No | BMC00827 |
| L12 | XP_033467.1 | CG3195 | JC8.3 (IV) | No | BMC01716 |
| L13 | XP_047464.1 | AAF52842.1 | C32E8.2 (I) | Yes | BMC00623 |
| L13a | XP_027886.1 | CG1475 | M01F1.2 (III) | Yes | BMC00137 |
| L14 | XP_044190.1 | AAF50393.1 | C04F12.4 (I) | No | BMC00082 |
| L15 | XP_048417.1 | AAF45440.1 | F10B5.1 (II) | Yes | BMC00009 |
| L17, L23 | XP_028962.1 | AAF46914.1 | B0336.10 (III) | Yes | BMC00275 |
| L18 | XP_049965.1 | CG8615 | Y45F10D.12 (IV) | Yes | BMC00862 |
| L18a | XP_038594.1 | AAF57838.1 | E04A4.8 (IV) | No | BMC00060 |
| L19 | XP_008294.3 | AAF47305.1 | C09D4.5 (I) | Yes | BMC02351 |
| L21 | XP_033917.1 | CG12775 | C14B9.7 (III) | No | BMC01506 |
| L22 | XP_030989.1 | AAF45546.1 | C27A2.2 (II) | No | BMC03219 |
| L23 | XP_012891.1 | AAF47545.1 | F55D10.2 (X) | Yes | BMC00175 |
| L23a | XP_017356.1 | AAF47545.1 | F52B5.6 (I) | No | BMC00175 |
| L24 | XP_015463.1 | CG9282 | D1007.12 (I) | Yes | BMC00120 |
| L26 | XP_016869.1 | CG6846 | F28C6.7 (II) | Yes | BMC00059 |
| L27 | XP_032124.1 | CG4759 | C53H9.1 (I) | Yes | BMC03199 |
| L27a | XP_016869.1 | AAF51006.2 | Y37E3.8A (I) | Yes | BMC01540 |
| L28 | XP_035923.1 | CG12740 | R11D1.8 (V) | No | BMC01157 |
| L29 | XP_011055.1 | AAF46708.1 | B0513.3 (IV) | Yes | BMC00067 |
| L30 | XP_046141.1 | CG6764 | C03D6.8 (I) | No | BMC02758 |
| L31, L41 | XP_033301.1 | CG1821 | W09C5.6A/B (I) | No | BMC01624 |
| L32 | XP_003054.3 | AAF57001.1 | T24B8.1 (II) | No | BMC00221 |
| L34 | XP_034712.1 | CG6090 | C42C1.14 (IV) | No | BMC00060 |
| L35 | XP_044796.1 | CG4111 | ZK652.4 (III) | No | BMC00205 |
| L35a | X52966 | CG2099 | F10E7.7 (II) | No | BMC00179 |
| L36 | XP_044614.1 | AAF45531.1 | F37C12.4 (III) | Yes | BMC00060 |
| L36a | XP_052671.1 | CG7424 | C09H10.2 (II) | No | BMC01314 |
| L37 | XP_017770.1 | CG9091 | W01D2.1 (II) | No | BMC00060 |
|  | XP_017770.1 | CG9091 | C54C6.1 (III) | No | BMC00060 |
| L37a | NP_000989 | CG5827 | Y48B6A.2 (II) | Yes | BMC01949 |
| L38 | P23411 | CG18001 | C06B8.8 (V) | Yes | BMC00157 |
| L39 | XP_010359.3 | AAF47154.1 | [C26F1.9](http://www.ncbi.nlm.nih.gov/htbin-post/Entrez/query?form=6&dopt=g&db=p&uid=01710550) (V) | No | BMC00809 |
| L40 | XP_009284.3 | AAF51034.1 | ZK1010.1 (III) | Yes | BMC00134 |

**Supplementary Materials, Table S3b:** Small subunit cytoplasmic ribosomal proteins

| **protein name** | ***H. sapiens*** | ***D. melanogaster*** | ***C. elegans*** | **Operonic in *C. elegans*** | ***B. malayi*** |
| --- | --- | --- | --- | --- | --- |
| S2 | XP_043619 | AAF45638.1 | B0393.1 (III) | No | BMC00957 |
| S2/S5 | XM_034464 | AAF52822.1 | C49H3.11 (IV) | Yes | BMC01616 |
| S3 | XP_035076.1 | AAF56129.1 | C23G10.3 (III) | Yes | BMC00419 |
| S3a | XP_037456.1 | AAF59372.1 | F56F3.5 (II) | No | BMC01505 |
| S4 | XP_044025.1 | AE003539 | Y43B11AR.4 (IV) | Yes | BMC00375 |
| S5 | XP_034265 | CG7014 | T05E11.1 (IV) | Yes | BMC00499 |
| S6 | XP_048310.1 | AAF46288.1 | Y71A12B.1 (I) | No | BMC00011 |
| S7 | XP_012638.5 | CG1883 | ZC434.2 (I) | No | BMC00849 |
| S8 | XP_046554.1 | CG7808 | F42C5.8 (IV) | No | BMC00738 |
| S9 | XP_050590.1 | AAF50249.1 | F40F8.10 (II) | No | BMC01891 |
| S10 | XP_043285.1 | CG14206 | D1007.6 (I) | Yes | BMC00256 |
| S11 | P04643 | AAF50249.1 | F40F11.1 (IV) | No | not found |
| S12 | XP_017626.1 | AAF49851.1 | F54E7.2 (III) | Yes | BMC00188 |
| S13 | XP_047325.1 | CG4263 | C16A3.8 (III) | No | not found |
| S14 | XP_042550.1 | AAF46297.1 | F37C12.9 (III) | No | BMC00207 |
| S15 | XP_047576.1 | CG8332 | F36A2.6 (I) | No | BMC04325 |
| S15a | XP_027366.1 | CG2033 | F53A3.3 (III) | No | BMC00121 |
| S16 | XP_046112.1 | CG4046 | T01C3.6 (V) | Yes | BMC00243 |
| S17 | XP_007615.3 | AAF50272.1 | T08B2.10 | Yes | BMC00267 |
| S18 | XP_016854.1 | AAF57491.1 | Y57G11C.16 (IV) | No | BMC00161 |
| S19 | XP_008876.1 | AAF48633.1 | T05F1.3 (I) | No | BMC00176 |
| S20, S22 | XP_031816.1 | AAF55809.1 | Y105E8A.16 (I) | Yes | BMC00239 |
| S21 | XP_009693.3 | AAF51191.1 | F37C12.11 (III) | No | BMC00282 |
| S23 | XP_004020.1 | CG8415 | F28D1.7 (IV) | No | BMC00817 |
| S24 | XP_039577.1 | CG3751 | T07A9.11 (IV) | No | BMC00096 |
| S24e | XP_039578.1 | not found | [T26G10.3](http://www.ncbi.nlm.nih.gov/htbin-post/Entrez/query?form=6&dopt=g&db=p&uid=00465977) (III) | No | not found |
| S25 | XP_051497.1 | AAF54605.1 | [K02B2.5](http://www.ncbi.nlm.nih.gov/htbin-post/Entrez/query?form=6&dopt=g&db=p&uid=01710752) (IV) | Yes | BMC00290 |
| S26 | XP_049421.1 | AAF53666.1 | F39B2.6 (I)  C03H5.f (II) | No | BMC00253 |
| S27 | P42677 | CG8338 | F56F3.5 (III) | No | BMC01505 |
| S27a | XP_017513.2 | AAF52941.1 | F34H10.1 (X)  K08C9.7 (I) | No  No | BMC03367 |
| S28 | XP_006026.2 | CG2998 | Y41D4B.5 (IV) | No | BMC00209 |
| S29 | XP_052669.1 | CG8495 | [B0412.4](http://www.ncbi.nlm.nih.gov/htbin-post/Entrez/query?uid=gb|U80953|&form=6&db=n&Dopt=g) (III) | Yes | BMC00288 |
| S30 | XP_006522.3 | CG15697 | [C26F1.4](http://www.ncbi.nlm.nih.gov/htbin-post/Entrez/query?uid=gb|U53148|&form=6&db=n&Dopt=g) (V) | Yes | BMC00146 |
| P0 | XP_017620.1 | AAF51807.1 | F25H2.10 (I) | Yes | BMC00405 |
| P1 | XP_035388.1 | AAF51499.1 | Y37E3.7 (I) | Yes | BMC00166 |
| P2 | M17887 | R6FFP2 | C37A2.7 (I) | Yes | BMC00278 |
