# Supplementary material for: Operon Conservation and the Evolution of trans-Splicing in the Phylum Nematoda
Source: PLoS Genet. 2006 Nov 24;2(11):e198. doi: 10.1371/journal.pgen.0020198 (PMC1657053; doi:10.1371/journal.pgen.0020198)
Supplement: Table S4 — (158 KB DOC) [file pgen.0020198.st004.doc]

**Supplementary Materials, Table S4: Alternate SL usage identified in *Ascaris suum, Brugia malayi, Nippostrongylus brasiliensis, Strongyloides ratti,* and *Pristionchus pacificus***

**Supplementary Materials, Table S4a:** Variant Spliced leader 1 sequences isolated from *A. suum, B. malayi, N. brasiliensis,* and *P. pacificus*

|  | Number of clones | Percent of SL-1 dataset | Found in species |
| --- | --- | --- | --- |
| SL1-like sequences | | | |
| GGTTT-AATTACCCAAGTTTGAG | 237 | 97% | all |
| GGGTTTAATTACCCAAGTTTGAG | 2 | <1% | *B. malayi* *, P. pacificus* |
| GGTTT-AATCACCCAAGTTTGAG | 1 | <1% | *B. malayi* |
| GGTTT-AACTACCCAAGTTTGAG | 2 | <1% | *B. malayi, A. suum* |
| GGTTT-AATTGCCCAAGTTTGAG | 1 | <1% | *A. suum* |
| GGTTT-AATAACCCAAGTTTGAG | 1 | <1% | *N. brasiliensis* |

**Supplementary Materials, Table S4b:** Spliced leader sequences isolated from *P. pacificus* (5’ ends of *Pp-rpa-1*)

|  | Number of clones | Percent of dataset | Found in other species |
| --- | --- | --- | --- |
| SL1-like sequences | | | |
| GGTTT-AATTACCCAAGTTTGAG | 7 | 4% | all |
| GGTTTTAATTACCCAAGTTTGAG | 1 | <1% | *B. malayi* |
| SL2-like sequences | | | |
| GGTTT-TAACCCAGTATCTCAAG | 106 | 62% | *H. contortus* |
| GGTTT-ATACCCAGTATCTCAAG | 23 | 13% |  |
| GGTTT-TTACCCAGTATCTCAAG | 21 | 12% | *O. tipulae* |
| GGTTT-AAACCCAGTATCTCAAG | 2 | 1% |  |
| GGTTTTTAACCCAGTATCTCAAG | 2 | 1% |  |
| GGGTTTTTACCCAGTATCTCAAG | 1 | <1% |  |
| GGGTTTATACCCAGTATCTCAAG | 1 | <1% |  |
| GGTTT-TTACTCAGTATCTCAAG | 1 | <1% |  |
| GGTCT-TTACCCAGTATCTCAAG | 1 | <1% |  |
| GGTTT-TAACCCGGTATCTCAAG | 1 | <1% |  |
| GGTTT-TAACCCAGTATCTTAAG | 1 | <1% |  |
| GGTTT-TGACCCAGTATCTCAAG | 1 | <1% |  |
| Total of SL sequences assayed | 161 |  |  |

**Supplementary Materials, Table S4c:** Spliced leader sequences isolated from *N. brasiliensis* (5’ ends of *Nb-rpl-27a*, *Nb-rpa-1*, *Nb-rpa-0*, and *Nb-tct-1*)

| SL sequence | Number of clones | % | Number of clones | % | Number of clones | % | Number of clones | % | Percent of dataset |
| --- | --- | --- | --- | --- | --- | --- | --- | --- | --- |
|  | *Nb-rpl-27a* |  | *Nb-rpa-1* |  | *Nb-rpa-0* |  | *Nb-tct-1* |  |  |
| SL1-like sequences | | | | | | | | | |
| GGTTTAATTACCCAAGTTT---GAG | 11 | 100% | 5 | 31% | 10 | 99% | 18 | 85% | 74% |
| GGTTTAATAACCCAAGTTT---GAG | 0 | 0 | 0 | 0 | 1 | 1% | 0 | 0 | <1% |
| SL2-like sequences | | | | | | | | | |
| GGT-AATTAACCC-AGTATCTCAAG | 0 | 0 | 6 | 46% | 0 | 0 | 2 | 10% | 13% |
| GGT-AATTA-CCC-AGTATCTCAAG | 0 | 0 | 2 | 13% | 0 | 0 | 0 | 0 | 3% |
| GGTT-AATA-CCC-AGTATCTCAAG | 0 | 0 | 1 | <1% | 0 | 0 | 0 | 0 | <1% |
| GGTTTAAA--CCC-AGTATCTCAAG | 0 | 0 | 1 | <1% | 0 | 0 | 0 | 0 | <1% |
| GGTTTTTA—-CCC-GGTATCTTAAG | 0 | 0 | 1 | <1% | 0 | 0 | 0 | 0 | <1% |
| GGT-AATTAACCA-AGTATCTCAAG | 0 | 0 | 0 | 0 | 0 | 0 | 1 | 5% | <1% |
| Total of SL sequences assayed | 11 |  | 16 |  | 11 |  | 21 |  | 59 |

**Supplementary Masterials, Table S4d:** Spliced leader sequences isolated from *S. ratti* (5’ ends of *Sr-rp1-27a,* *Sr-rpa-1, Sr-rpa-0*, *Sr-tct-1* and Sr-Y82E9BR.3)

| SL sequence | Number of clones | % | Number of clones | % | Number of clones | % | Number of clones | % | Number of clones | % | % across all genes |
| --- | --- | --- | --- | --- | --- | --- | --- | --- | --- | --- | --- |
|  | *Sr-rpl-27a* |  | *Sr-rpa-1* |  | *Sr-rpa-0* |  | *Sr-tct-1* |  | *Sr-*Y82E9BR.3 |  |  |
| GGTTT--AATTACCCAAGTT-TGAG | 0 | 0 | 0 | 0 | 0 | 0 | 0 | 0 | 0 | 0 | 0 |
| GGTTT--ATAAAACCCAGTT-TGAG | 5 | 50% | 45 | 52% | 6 | 60% | 4 | 40% | 43 | 40% | 46% |
| GGTTT--AAAAAACCCAGTT-TGAG | 2 | 20% | 24 | 26% | 2 | 20% | 1 | 10% | 48 | 44% | 34% |
| GGTTT---AAAAACCCAGTT-TGAG | 0 | 0 | 8 | 9% | 0 | 0 | 0 | 0 | 2 | 2% | 4% |
| GGTTT---TAAAACCCAGTT-TGAG | 0 | 0 | 3 | 3% | 0 | 0 | 0 | 0 | 0 | 0 | 1% |
| GGTTT---AAAAACCCAATT-TGAG | 0 | 0 | 1 | 1% | 0 | 0 | 0 | 0 | 0 | 0 | <1% |
| GGTTT--AAATAACCCAGTT-TGAG | 0 | 0 | 1 | 0 | 0 | 0 | 0 | 0 | 0 | 0 | <1% |
| GGTTT--AAATAACCCATAT-AGAG | 1 | 10% | 1 | 1% | 2 | 20% | 0 | 0 | 0 | 0 | 2% |
| GTTTTTTAAATAACCAAGTT-TGAG | 0 | 0 | 1 | 1% | 0 | 0 | 0 | 0 | 0 | 0 | <1% |
| GGTTT-AAGAAAAACCCATT-CAAG | 1 | 10% | 1 | 1% | 0 | 0 | 2 | 20% | 0 | 0 | 2% |
| GGTTT-TATAAAACCCAGTT-TGAG | 0 | 0 | 1 | 1% | 0 | 0 | 0 | 0 | 0 | 0 | <1% |
| GGTTT--AAAAAACCCAGTT-TAAG | 0 | 0 | 0 | 0 | 0 | 0 | 1 | 10% | 0 | 0 | <1% |
| GGTTT--ATAAAACCCAGTT-TAAG | 0 | 0 | 1 | 1% | 0 | 0 | 0 | 0 | 0 | 0 | <1% |
| GGTTT---AAAAACCCGATTTTGAG | 1 | 10% | 0 | 0 | 0 | 0 | 2 | 20% | 4 | 4% | 3% |
| GGTTT-TAAATAACCCAGTT-TGAG | 0 | 0 | 0 | 0 | 0 | 0 | 0 | 0 | 3 | 3% | 1% |
| GGTTT--ATATAACCCAGTT-TGAG | 0 | 0 | 0 | 0 | 0 | 0 | 0 | 0 | 2 | 2% | 1% |
| GGTTT---AAAAACCCAAAT-TAAA | 0 | 0 | 0 | 0 | 0 | 0 | 0 | 0 | 2 | 2% | 1% |
| GGTTT--TAAAAACCCAGTT-TGAG | 0 | 0 | 0 | 0 | 0 | 0 | 0 | 0 | 1 | 1% | <1% |
| GGTTT--ATACAACCCAGTT-TGAG | 0 | 0 | 0 | 0 | 0 | 0 | 0 | 0 | 1 | 1% | <1% |
| GGTTT--AAGAAACCCTGTT-TGAG | 0 | 0 | 0 | 0 | 0 | 0 | 0 | 0 | 1 | 1% | <1% |
|  | 10 |  | 87 |  | 10 |  | 10 |  | 107 |  | 224 |
