# Supplementary material for: Operon Conservation and the Evolution of trans-Splicing in the Phylum Nematoda
Source: PLoS Genet. 2006 Nov 24;2(11):e198. doi: 10.1371/journal.pgen.0020198 (PMC1657053; doi:10.1371/journal.pgen.0020198)
Supplement: Table S5 — (75 KB DOC) [file pgen.0020198.st005.doc]

**Supplementary Materials, Table S5: Conserved operons in species other than *Caenorhabditis elegans***

These tables list the size of the genes predicted from the cloned genomic fragments, the size of the introns within each gene, and the intergenic distance between the two operonic genes. ND: No data available.

**Supplementary Materials, Table S5a:** OP1032 (*rpl-27a* / *rpa-1*)

| Species | *rpl2-7a* gene/ gene fragment size in bp | *rpl-27a* intron sizes in bp | Intergenic region size in bp | *rpa-1*gene/ gene fragment size in bp | *rpa-1* intron sizes in bp |
| --- | --- | --- | --- | --- | --- |
| *C. elegans* | 1106 | 215, 233 | 104 | 488 | 50 |
| *O. tipulae* | 596 | 81, 42 | 89 | 544 | 39, 68 |
| *P. pacificus* | 631 | 74, 96 | 89 | 281 | 69 |
| *N. brasiliensis* | 1548 | 637, 469 | 141 | 634 | 233 |
| *S. ratti* | 530 | 48 | 130 | 312 | 43 |
| *B. malayi* | 989 | 425 | 272 | 648 | 410 |
| *A.suum* | 2752 | 2308 | 802 | 862 | 466 |

**Supplementary Materials, Table S5b:** OP1624 (*rpa-0* / *tct-1*)

| Species | *rpa-0* gene/ gene fragment size in bp | *rpa-0* intron sizes in bp | Intergenic region size in bp | *tct-1* gene/gene fragment size in bp | *tct-1* intron sizes in bp |
| --- | --- | --- | --- | --- | --- |
| *C. elegans* | 1121 | 47, 46 | 104 | 902 | 256 |
| *P. pacificus* | 608 | - | 95 | 877 | 269, 76 |
| *N. brasiliensis* | 820 | 67 | 506 | 743 | 289, 69 |
| *S. ratti* | 1066 | 48 | 157 | 744 | 275 |
| *B. malayi* | 749 | 151, 102 | 394 | 1379 | 521, 151, 176 |
| *A.suum* | 1255 | 410,266 | 838 | 1962 | 750, 263, 422 |

**Supplementary Materials, Table S5c:** OP3416 (*rpl-36* / F37C12.3)

| Species | *rpl-36* gene/ gene fragment size in bp | *rpl-36* intron sizes in bp | Intergenic region size in bp | F37C12.3 gene/gene fragment size in bp | F37C12.3 intron sizes in bp |
| --- | --- | --- | --- | --- | --- |
| *C. elegans* | 427 | 51 | 75 | 1155 | 201, 46, 336 |
| *P. pacificus* | 1485 | 1102 | 347 | 816 | 288, 138 |
| *S. ratti* | 378 | 43 | ND | ND | ND |
| *B. malayi* | 566 | 104, 92 | 405 | 572 | 103 |

**Supplementary Materials, Table S5d:** OP5428 (*fib-1* / *rps-16*)

| Species | *fib-1* gene/ gene fragment size in bp | *fib-1* intron sizes in bp | Intergenic region size in bp | *rps-16* gene/gene fragment size in bp | *rps-16* intron sizes in bp |
| --- | --- | --- | --- | --- | --- |
| *C. elegans* | 1309 | 448, 48, 49 | 120 | 515 | 45 |
| *P. pacificus* | 833 | 67, 229 | 110 | 505 | 66 |
